# Genome-wide CNV analysis reveals variants associated with growth traits in *Bos indicus*

**DOI:** 10.1186/s12864-016-2461-4

**Published:** 2016-06-01

**Authors:** Yang Zhou, Yuri T. Utsunomiya, Lingyang Xu, El Hamidi abdel Hay, Derek M. Bickhart, Pamela Almeida Alexandre, Benjamin D. Rosen, Steven G. Schroeder, Roberto Carvalheiro, Haroldo Henrique de Rezende Neves, Tad S. Sonstegard, Curtis P. Van Tassell, José Bento Sterman Ferraz, Heidge Fukumasu, Jose Fernando Garcia, George E. Liu

**Affiliations:** Animal Genomics and Improvement Laboratory, BARC, USDA-ARS, Room 111, BARC-East, Beltsville, Maryland 20705 USA; College of Animal Science and Technology, Northwest A&F University, Shaanxi Key Laboratory of Agricultural Molecular Biology, Yangling, Shaanxi 712100 China; Departamento de Medicina Veterinária Preventiva e Reprodução Animal, Faculdade de Ciências Agrárias e Veterinárias, UNESP - Univ Estadual Paulista, Jaboticabal, São Paulo 14884-900 Brazil; Institute of Animal Science, Chinese Academy of Agricultural Science, Beijing, 100193 China; Department of Veterinary Medicine, College of Animal Science and Food Engineering, University of São Paulo, Pirassununga, SP 13635 Brazil; Departamento de Zootecnia, Faculdade de Ciências Agrárias e Veterinárias, UNESP - Univ Estadual Paulista, Jaboticabal, São Paulo 14884-900 Brazil; Departamento de Apoio, Produção e Saúde Animal, Faculdade de Medicina Veterinária de Araçatuba, UNESP – Univ Estadual Paulista, Araçatuba, São Paulo 16050-680 Brazil; Present address: Recombinetics, Inc., St Paul, MN 55104 USA; International Atomic Energy Agency (IAEA) Collaborating Centre on Animal Genomics and Bioinformatics, Araçatuba, SP Brazil

**Keywords:** Copy number variation (CNV), EBV, Association, Body size, *Bos indicus*

## Abstract

**Background:**

Apart from single nucleotide polymorphism (SNP), copy number variation (CNV) is another important type of genetic variation, which may affect growth traits and play key roles for the production of beef cattle. To date, no genome-wide association study (GWAS) for CNV and body traits in beef cattle has been reported, so the present study aimed to investigate this type of association in one of the most important cattle subspecies: *Bos indicus* (Nellore breed).

**Results:**

We have used intensity data from over 700,000 SNP probes across the bovine genome to detect common CNVs in a sample of 2230 Nellore cattle, and performed GWAS between the detected CNVs and nine growth traits. After filtering for frequency and length, a total of 231 CNVs ranging from 894 bp to 4,855,088 bp were kept and tested as predictors for each growth trait using linear regression analysis with principal components correction. There were 49 significant associations identified among 17 CNVs and seven body traits after false discovery rate correction (P < 0.05). Among the 17 CNVs, three were significant or marginally significant for all the traits. We have compared the locations of associated CNVs with quantitative trait locus and the RefGene database, and found two sets of 9 CNVs overlapping with either known QTLs or genes, respectively. The gene overlapping with CNV100, *KCNJ12*, is a functional candidate for muscle development and plays critical roles in muscling traits.

**Conclusion:**

This study presents the first CNV-based GWAS of growth traits using high density SNP microarray data in cattle. We detected 17 CNVs significantly associated with seven growth traits and one of them (CNV100) may be involved in growth traits through *KCNJ12*.

**Electronic supplementary material:**

The online version of this article (doi:10.1186/s12864-016-2461-4) contains supplementary material, which is available to authorized users.

## Background

Growth traits can directly affect production of beef cattle, which is an economically important sector of global agriculture. Both body weight measurements and visual trait scores of conformation, precocity and muscling are used to select calves and steers for breeding and to predict carcass parameters. Various factors can regulate growth traits through the modulation of gene expression levels, and genomic variation is one of the mechanisms, which is not only persistent but also inherited. To date, several genomic variations, such as STR (short tandem repeat, or microsatellite) and SNP (single nucleotide polymorphism) have been employed to identify genetic markers related to cattle growth traits [1, 2]. Copy number variation (CNV) belongs to another family of genetic markers, which could affect gene expression and consequently phenotypes by changes in gene structure and dosage [3]. In human and mouse studies, CNV was found to account for 18 to 30 % of the genetic variation in gene expression and showed critical roles in both normal phenotypic variability and disease susceptibility [4, 5]. However, only few studies have been published using CNVs as candidate markers for genetic selection and disease association at the genome level because the traditional CNV discovery studies tried to detect as many variable regions as possible, instead of emphasizing common CNVs shared by different individuals [6].

The application of CNV information in animal breeding had long been held back due to the lack of reliable genotyping methods for CNVs. In livestock, most CNV studies had been limited to CNV detection methods [2, 7–10]. Studies performing CNV association with body traits were mainly focused on single rather than genome-wide CNVs [11–13]. Several studies attempted to predict the effects of CNVs on phenotypes based on the function of genes within the CNV regions. For example, an earlier study in Holstein cattle reported CNV loci enriched for solute carriers and ABC transporters that may affect milk production traits [14]. Exploration of genome-wide association study (GWAS) using CNVs and phenotypes has just gotten started recently. Our previous studies have indicated that CNVs could be associated with resistance or susceptibility to gastrointestinal nematodes in Angus cattle [15] and residual feed intake in Holstein cows [16]. A study on Chinese cattle has shown that one CNV region (CNVR) had significantly negative effects on expression of *PLA2G2D* gene and two CNVRs were associated with body measurements [17]. Subsequently, we have reported and characterized 34 significantly associated CNVs with milk production traits in Holsteins [6] and found one deletion polymorphism associated with resistance to gastrointestinal nematodes in Angus cattle [18]. The increase in resolution of the SNP microarray data dramatically improves the accuracy and the number of CNV detected. Up to now, no systematic study of the relationship between CNVs and beef growth traits using SNP microarray data with high resolution has been published.

Few CNV studies including *Bos indicus* data have been reported. We once performed a high resolution CNV analysis in 674 animals of 27 breeds, which included 113 animals with *Bos indicus* background [19]. *Nellore* is one the most important sub-breeds of *Bos indicus* cattle, which is predominant in Brazil, the world’s second largest producer of cattle. They are farmed for beef that can be seriously affected by growth traits. The present study is the first using common CNVs to detect association with production traits in *Bos indicus*. Using BovineHD Genotyping BeadChip assay data of 2230 Nellore bulls and cows, we detected common CNVs and performed a systematic GWAS analysis between CNVs and nine growth traits in order to identify candidate CNVs and genes that could be used in cattle genetic improvement programs.

## Results and Discussion

### CNV segments and genotyping

**Fig. 1:**
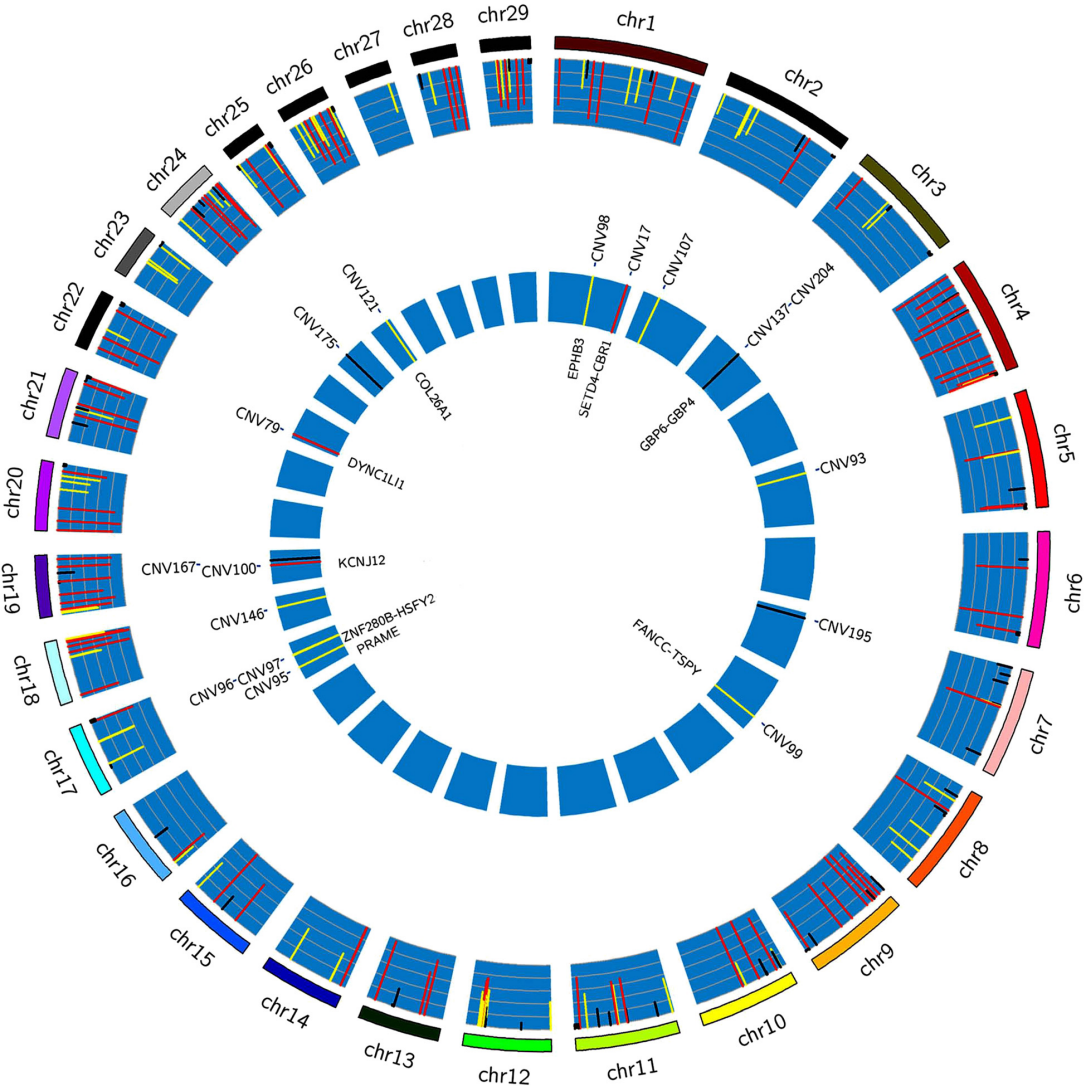
Distribution of the high confidence CNVs and significantly associated CNVs on each chromosome. Outside circle: distribution of the high confidence CNVs, the height of histograms represents the variant frequency of each CNV; Inside circle: distribution of the significantly associated CNVs and its overlapped genes; Colors of the histograms for variant frequency: red, over 0.6; yellow, between 0.3 and 0.6; black, under 0.3

In total, 992,350 CNVs were extracted by the multivariate method supplied by Golden Helix SVS 8.3.0. After merging all the segments, we found 445 non-redundant CNV events (copy loss, neutral and copy gain) occurring in the 2230 individuals analyzed. As generally accepted, a CNV is one type of structural variation ranging from 50 bp to 5 Mb [20, 21]. We used a two-step strict method to filter possibly false CNVs. First, we calculated the frequencies of the three CNV types and filtered those appearing in less than 10 samples (0.45 %). Then, CNVs over 5 Mb were filtered out. In fact, when we filtered CNVs with low frequency, there were only two CNVs over 5 Mb with frequencies of 0.45 %. This meant that large segments were mainly predominant with low frequency and could be removed by a high frequency threshold. Finally, a total of 231 CNVs with high confidence, ranging from 894 bp to 4,855,088 bp, were retained and used in further analysis. They were named as CNV1 to CNV231, according to their loss frequencies from highest to lowest (Fig. 1 and Additional file 1: Table S1). We performed 45 quantitative PCRs to validate 5 selected CNVs in 9 samples and over 80 % of the results were in agreement with our SVS predictions (Additional file 1: Table S2).

### Traits’ correlation analysis

**Fig. 2:**
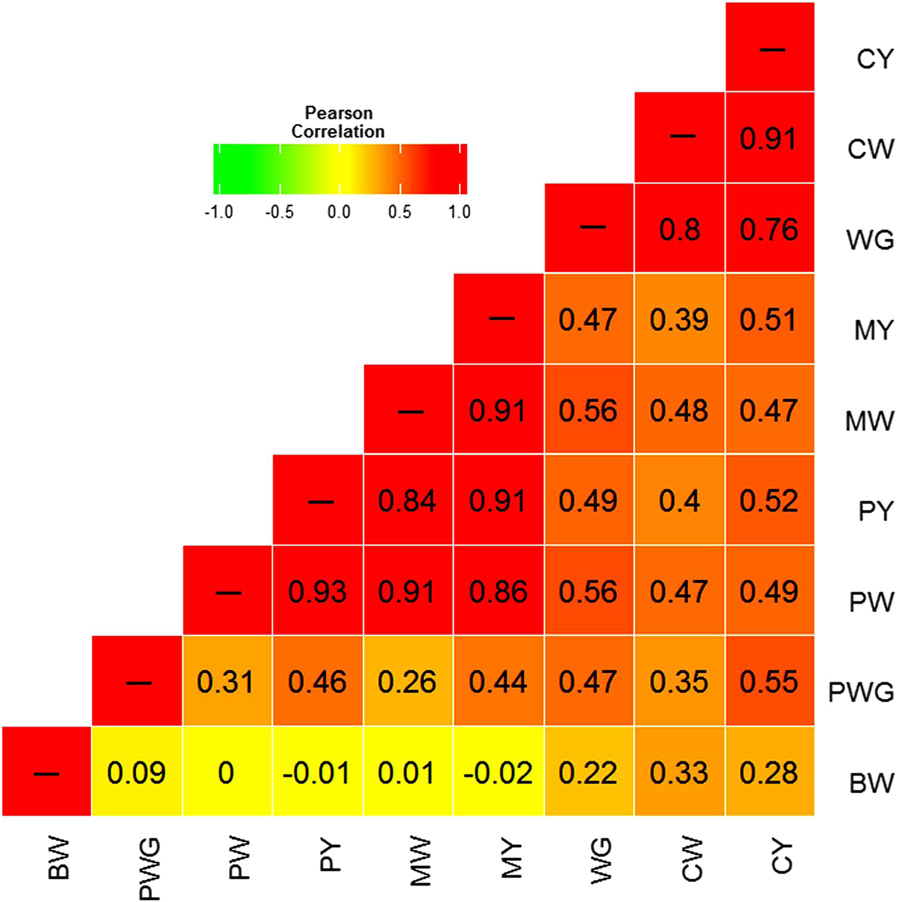
Pairwise Pearson correlation coefficients for the nine body traits

Body weight measurements and visual scores are important traits in the evaluation of carcass yield for beef cattle selection in field conditions. In this study, nine related body traits were selected to be employed in further analysis: birth weight (BW), post weaning gain (PWG), weaning gain (WG), conformation at weaning (CW), muscling at weaning (MW), precocity at weaning (PW), conformation at yearling (CY), muscling at yearling (MY) and precocity at yearling (PY). Estimated breeding values (EBV) were available for all traits, and only samples with accuracy of at least 0.5 were analyzed for each trait. In order to assess whether these traits present genetic correlations, we computed Pearson product-moment correlation coefficients for all pairs of the nine traits from 2013 Nellore cattle with complete data (Fig. 2). The results showed that BW and PWG had low correlation (R = 0.09), and they were both positively correlated with WG showing R values of 0.22 and 0.47, respectively. Interestingly, the correlation of these body weight traits with the visual traits of conformation, precocity and muscling (CPM) exhibited regularly increasing trends along with the increasing age. BW had no or low correlation with visual traits. On the other hand, the body weight traits after birth (PWG and WG) had higher correlations with CPM, especially WG, which showed strong correlations with CW (0.8) and CY (0.76). As expected, the six CPM traits were all positively correlated with each other in our study. Precocity and muscling were strongly correlated with the lowest value of 0.84 between MW and PY. Lower correlations (R value from 0.39 to 0.52) were seen for conformation traits with the other four CPM traits. Since these traits were not independent, it is expected that certain CNVs may be associated with two or more related body traits and fewer significantly associated CNVs would be found than expected as compared to the situation where the nine traits were independent. Moreover, as these correlations suggest genetic dependence among traits, CNVs regulating two or more traits may be considered as pleiotropic structural variants.

### CNV significant association analysis

**Fig. 3:**
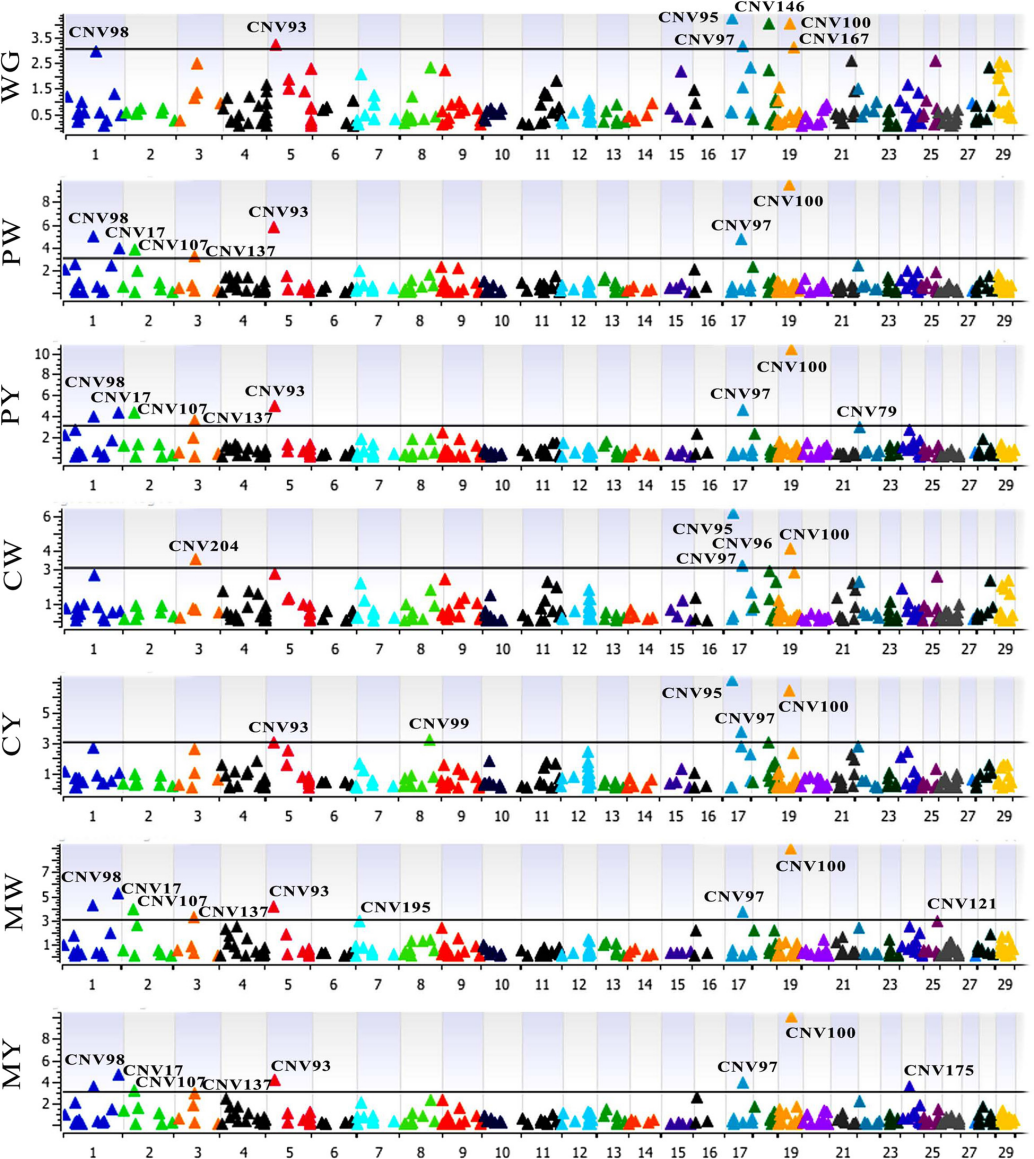
Manhattan plots of associated CNVs for WG and CPM traits. Negative log 10-transformed P values from a genome-wide scan were plotted against genomic coordinates on 29 autosomal chromosomes

It is difficult to directly use the traditional CNV discovery method in CNV-based GWAS since they tend to expand CNV counts instead of finding common CNVs [6]. In this study, we performed a genome wide association analysis using the 231 CNVs and three body weight traits plus six visual (CPM) traits. There were 49 significant associations identified among 17 CNVs and 7 body traits. No significantly associated CNVs were found for BW and PWG. The lowest P value for BW was 0.431. CNV153 and CNV66 had trends to be associated with PWG, with P values of 0.063 and 0.096 respectively. For the other seven traits, we found 7, 5, 5, 9, 8, 7 and 8 CNVs significantly associated with WG, CW, CY, MW, MY, PW and PY, respectively (Fig. 3 and Table 1). Among the 17 CNVs, two CNVs (CNV97 and CNV100) were significantly associated with all 7 traits, and seven CNVs (CNV17, CNV93, CNV97, CNV98, CNV100, CNV107 and CNV137) were commonly associated with all of the precocity and muscling traits. CNV93 had trends to be related to CW (0.075) and it was related to all other six traits with significance. Additionally, only nine CNVs had their unique associated body traits. Those results were consistent with the trait correlation results.

**Table 1:** Characterization of the association CNVs

CNV	Supported by		*P* value after FDR correction							Overlap with		
	PennCNV	HapMap	WG	PW	PY	CW	CY	MW	MY	Gene symbol	QTL ID	QTL phenotype
CNV17	Yes	Yes	0.6589	**0.0068**	**0.0036**	0.8175	0.5368	**0.0010**	**0.0035**	*SETD4, CBR1*		
CNV79	Yes	No	0.2911	0.1268	**0.0450**	0.1043	0.0580	0.0826	0.1497	*DYNC1LI1*		
CNV93	Yes	Yes	**0.0438**	**0.0002**	**0.0017**	0.0752	**0.0484**	**0.0054**	**0.0063**		22866	Intramuscular fat
CNV95	Yes	Yes	**0.0157**	0.8172	0.8042	**0.0002**	**0.0000**	0.8961	0.8287	*PRAME*		
CNV96	Yes	Yes	0.2910	0.9430	0.8805	**0.0382**	0.0643	0.9529	1.0000	*ZNF280B, HSFY2*	19024	Clinical mastitis
CNV97	Yes	Yes	**0.0376**	**0.0015**	**0.0027**	**0.0453**	**0.0191**	**0.0089**	**0.0081**		19024	Clinical mastitis
CNV98	Yes	Yes	**0.0439**	**0.0011**	**0.0052**	0.0789	0.0637	**0.0057**	**0.0155**	*EPHB3*		
CNV99	Yes	No	0.0927	0.3515	0.3329	0.1864	0.0491	0.3428	0.1107	*FANCC, TSPY*		
CNV100	No	No	**0.0093**	**0.0000**	**0.0000**	**0.0109**	**0.0001**	**0.0000**	**0.0000**	*KCNJ12*	22873	Intramuscular fat
CNV107	Yes	No	0.6665	**0.0074**	**0.0033**	0.9921	0.9579	**0.0064**	**0.0315**		20642	Iron content
CNV121	Yes	No	0.0918	0.2194	0.5162	0.0784	0.3762	**0.0379**	0.4378	*COL26A1*	15219	Calf size
CNV137	Yes	Yes	0.3419	**0.0276**	**0.0126**	0.8045	0.5354	**0.0211**	**0.0434**			
CNV146	Yes	Yes	**0.0132**	0.9858	0.8931	0.0663	0.0546	0.9304	0.8783			
CNV167	No	No	**0.0390**	0.7426	0.8412	0.0674	0.1045	0.8952	0.9391		22873	Intramuscular fat
CNV175	Yes	Yes	0.2494	0.1736	0.0704	0.5176	0.0921	0.0772	**0.0160**			
CNV195	Yes	Yes	0.1181	0.1798	0.2897	0.1032	0.2477	**0.0371**	0.1585		15419	Gestation length
CNV204	Yes	Yes	0.0892	0.9530	1.0000	**0.0270**	0.0712	0.9434	1.0000	*GBP6, GBP4*		

Note: PennCNV: We applied PennCNV analysis using the same samples. HapMap: The previous study using the Bovine HD microarray data from 27 cattle breeds [18]

### Significant CNV characterization

We compared the 17 significantly associated CNVs discovered with those previously described in Hou’s study with the same cattle genome reference [19]. Only 6 CNVs were not supported by Hou’s study. The frequencies of the 17 CNVs ranged from 8.25 % to 95.65 %, with the lowest frequency CNV204 located at chr3:54,541,000-54,963,401 and the highest frequency CNV17 located at chr1:150,048,097-150,057,730. The longest CNV was also CNV204 with 422,402 bp and the shortest CNV175 (chr24: 24,432,820-24,434,451) was 1632 bp. To further analyze the association between discovered CNVs and body traits, we overlapped the 17 significant CNVs with previously reported QTL at the Cattle QTL database (http://www.animalgenome.org/cgi-bin/QTLdb/BT/index). Considering all together, 8 CNVs were found to be overlapped with QTLs (Table 1). There were three CNVs (CNV93, CNV100 and CNV167) overlapped with QTLs that regulate intramuscular fat which is highly related to the growth traits. Iron content is an important factor affecting the quality of beef, including color and flavor. We found CNV107 significantly associated with MW, overlapping with QTLs related to iron content, suggesting that there may be some relations between iron content and MW. It is noted that although we used comparatively strict criteria to examine the overlap of CNV with QTL, some of them may be due to chance.

CNVs can have dramatic phenotypic consequences caused by altering gene dosage, disrupting gene coding sequence or perturbing long-range gene regulation. We compared the locations of the 17 significantly associated CNVs with the bovine refGene database (http://hgdownload.soe.ucsc.edu/goldenPath/bosTau6/database/). There were 9 CNVs overlapping with 13 genes (Fig. 1 and Table 1). Among these 13 genes, five (*KCNJ12*, *SETD4*, *CBR1*, *DYNC1LI1* and *PRAME*) were overlapped by at least one exon on their 5’ terminal end. CNV98 and CNV121 were completely located at the last introns of *EPHB3* and *COL26A*, respectively. Conversely, five genes were completely located within two CNV regions (*GBP6* and *GBP4* in CNV204, *HSFY2* and *ZNF280B* in CNV96, *TSPY* in CNV99). CNV99 was also overlapped with the 9th intron of *PANCC*. Nucleotide sequence variation in the promoter region can also affect the corresponding gene’s expression. We also examined whether any CNVs overlapped with regions up to 2000 bp upstream of gene transcript start sites but none was found.

### *KCNJ12* as a candidate gene for muscling

Several of the genes found in this study might contribute to body traits, when associated with overlapped CNVs, according to their Gene Ontology and pathway annotation in different species. We selected *KCNJ12* (potassium inwardly-rectifying channel, subfamily J, member 12), located at chr19: 35,955,280-35,993,796 as a candidate to do further analysis. Its first exon overlaps with CNV100 (chr19: 35933815-35957534), which was significantly associated with the two highly related traits (muscling and precocity), as well as being associated with the WG and the visual conformation traits. Both muscling and precocity visual traits are designed to predict carcass features in the live animal, as an approximation of the muscle (meat) contained in the carcass itself after slaughtering. Previous studies have shown that *KCNJ12* gene family members were expressed in brain and muscle [22]. RNA-seq analysis reported by previous studies revealed that bovine *KCNJ12* gene expression was significantly up-regulated from fetal to adult stage in longissimus muscle [23], pointing out its role in muscle development.

However, to date, there is little knowledge about this gene in cattle. We first compared the amino acids sequence of *KCNJ12* in different species including cattle, human, mouse, rat, monkey, pig and dog, showing that *KCNJ12* was fairly conserved except for some variable amino acids in the 3’ termination among all the mammals (Additional file 2: Figure S1). This means that the function of *KCNJ12* gene may also be conserved in the above species. According to the Gene Ontology Annotation (UniProt-GOA) Database, the cattle *KCNJ12* gene has 9 GO terms and it modulates the activity of inward rectifier potassium channel, which regulates the electrical activity of neuronal cells, insulin secretion and epithelial K+ transport [24]. Since the muscle fiber number does not increase after birth [25], the increase of muscle mass is mainly due to increase in length and cross-sectional area of muscle fibers stimulated by neural, hormonal and nutritional factors after birth. In human, *KCNJ12* was found to participate in muscling contraction process [26]. This may be one of the mechanisms by which *KCNJ12* regulates the amount of muscle mass through the modulation of muscle contraction in cattle. *KCNJ12* was annotated in the GABAB receptor activation pathway, in which GABAB receptors (GABABR) are metabotropic transmembrane receptors for gamma-aminobutyric acid (GABA) which are linked via G-proteins to potassium channels. Previous studies reported that they were required for normal development and that, together with ionotropic GABAA receptors, GABABR could help GABA to stimulate feeding which is another mechanism to promote muscle or even fat accumulation [27, 28]. It’s worth mentioning that the CNV100 was also overlapped with QTLs related to intramuscular fat and calf size. Moreover, we checked the KCNJ12 expression pattern in Bovine Gene Atlas data (http://bovineatlas.arl.arizona.edu/) [29], hosted by the innatedb (http://www.innatedb.com/) [30]. We found that it was expressed in 45 out of 87 tissues. It was highly expressed in multiple muscle types or muscle related tissues (Additional file 2: Figure S3). We examined the effect of the CNV100’s copy number on the gene expression of *KCNJ12* in muscle tissues using qPCR. The Nellore cattle with 3 copies of CNV100 had a significantly higher RNA level of the *KCNJ12* gene than the Nellore cattle with 2 copies had (*p* < 0.05, ANOVA examination, Additional file 2: Figure S4). Therefore, *KCNJ12* might also help muscle accumulation by increasing food intake through cooperation with GABAB receptors. In summary, the bovine *KCNJ12* gene could be selected as a candidate gene for muscling traits in beef cattle in future studies.

## Conclusion

This is the first study applying GWAS approaches between the common CNVs and *Bos indicus* (Nellore breed) growth traits using bovine high density SNP microarray data. The use of high-density SNP panels enabled the increase in four times the number of CNVs found when compared to Holstein cattle using low density SNP microarray data [23]. We identified 231 CNVs with high confidence and 17 of them were significantly associated with seven moderately or highly correlated growth traits. Three CNVs (CNV93, CNV97 and CNV100) were found significantly associated with all the seven growth traits. Our association study was also supported by the results of overlapping the CNV locations with known QTLs and RefGene databases. We selected *KCNJ12*, overlapped with CNV100, to functionally assess and concluded that it may be a candidate gene for muscling through the modulation of muscle contraction and food intake.

## Methods

### Samples

A total of 952 Nellore bulls and 1278 Nellore cows were genotyped for 777,962 SNPs with the Illumina® BovineHD Genotyping BeadChip assay (Illumina Inc.; 2011 BovineHD Genotyping BeadChip Data Sheet: DNA Analysis. http://www.illumina.com/Documents/products/datasheets/datasheet_bovineHD.pdf), according to the manufacturer’s protocol. This dataset builds on the data reported by previous studies [2], and comprises part of the genomic selection reference population from a commercial breeding program (DeltaGen) ran by an alliance of Nellore cattle breeders from Brazil. Genotyping data is available upon request from co-corresponding authors for research purposes. All animal protocols were approved by the Institutional Animal Care and Use Committee of Faculty of Food Engineering and Animal Sciences, University of São Paulo (FZEA-USP – protocol number 14.1.636.74.1). Additionally, genomic DNA was extracted either from commercialized semen straws (bulls) or stored hair (cows) samples. To examine the effect of CNV100’s copy number on the gene expression of *KCNJ12*, DNA and RNA samples were collected from 24 Nellore cattle muscle tissues.

### Phenotypic and EBV values

Estimated breeding values (EBVs) were based on Best Linear Unbiased Predictor (BLUP) estimates of single-trait animal models obtained from routine genetic evaluations using performance and pedigree data from the database (available at: http://www.gensys.com.br/home/show_page.php?id=701). Phenotypes used to fit the models comprised records from 542,918 animals born between 1985 and 2011, and raised in 243 grazing-based herds. The evaluated traits included birth weight (BW), post weaning gain (PWG), weaning gain (WG), carcass conformation at weaning (CW), muscling at weaning (MW), carcass finishing precocity at weaning (PW), carcass conformation at yearling (CY), muscling at yearling (MY) and carcass finishing precocity at yearling (PY). Conformation, finishing precocity and muscling traits (CPM) were based on recorded visual scores assigned in a discrete ordered scale, relative to the animals of the same management group. For each trait, only EBVs of animals whose accuracy (i.e., square root of reliability, calculated based on prediction error variance estimates) was > 0.50 were analyzed (Additional file 2: Figure S2 and Additional file 1: Table S3).

### CNV segmentation and genotyping

The high intensity data generated by Illumina BovineHD Genotyping BeadChip assay was applied to detect the common CNVs shared by different Nellore cattle. After exporting the DSF file from GenomeStudio Software, we imported its Log R Ratios (LRR) into Golden Helix SNP & Variation Suite (SVS) 8.3.0 (Golden Helix Inc., Bozeman, MT, USA) and successfully mapped 753,182 SNPs (96.81 %) onto the 29 autosomes of *Bos taurus* genome assembly UMD 3.1. The LRR was then normalized using the default GC correlation file to correct the waviness caused by the GC content. To define the CNV segments, the multivariate method was employed with following thresholds: (1) Max 20 segments per 10 k markers; (2) at least 3 markers per segment; (3) max pairwise segment p-value = 0.005. The three state covariates with a comparatively strict threshold (segment mean ±0.4) was used to define the CNVs as three types (loss, neutral and gain events) across all the samples.

### PCA-corrected association testing

We used the linear regression in the additive genetic model to identify the CNVs significantly associated with nine body traits individually with 10,000 permutations. The model was as follow:

$$ {y}_i\kern0.5em =\kern0.5em {\displaystyle \sum_{\boldsymbol{j}=\mathbf{\mathsf{1}}}^n{x}_{ij}\kern0.5em {\beta}_j+{e}_i} $$

Where *y*_*i*_ is the EBV of the ith individual, *x*_*ij*_ is the CNV genotype of the ith individual, *β*_*j*_ is the CNV effect, n is the number of CNVs and *e*_*i*_ is the residual. To correct the batch effects/stratification, we selected the Principle Component Analysis (PCA) option. The significance level of (*P* < 0.05) after FDR correction was chose to define the significant CNVs [6].

### Overlapping with QTL and gene analysis

Gene content of cattle CNV regions was assessed using RefGene annotation file in UCSC database (http://hgdownload.soe.ucsc.edu/goldenPath/bosTau6/database/). QTL database was downloaded from animal QTL database (http://www.animalgenome.org/cgi-bin/QTLdb/index, on UMD3.1 database). Overlaps with genes were detected using R script and defined as at least one bp overlap. We filtered the QTLs with confidence interval (CI) over 30 Mb because of over large CIs for some QTLs and used strict threshold to define the overlap as at least 50 % of the CNV length were covered by QTLs [30].

### CNV validation by qPCR

Primers were designed for qPCR validation using the NCBI Primer-BLAST webtool (http://www.ncbi.nlm.nih.gov/tools/primer-blast/index.cgi?LINK_LOC=BlastHome). Primer information can be seen in (Additional file 1: Table S4). SYBR green chemistry was used to carry out qPCR in triplicate reactions of 25 μl. All reactions were amplified on a bioRad MyIQ thermocycler. A common Nellore sample was chosen as the reference for all qPCR experiments and the *BTF3* and *GAPDH* were used as the internal DNA and RNA control, respectively. We examined the effect of CNV100’s copy number on the expression of *KCNJ12*. The CNV100’s copy numbers were determined using qPCR and two samples were found to be 3 copies. We selected twelve samples with 2 copies and the two samples with 3 copies to compare the RNA levels of the *KCNJ12* gene in muscle. Each sample was tested three times. The 2^-ΔΔCT^ method was employed to analyze the qPCR result [20].

## Additional files

Additional file 1: Table S1. Detail information of the CNVs detected with high confidence. **Table S2.** CNV validation by qPCR. **Table S3.** Number of animals used in the association analysis for each traits. **Table S4.** Primers for qPCR and rtPCR validations. (XLSX 37 kb) Additional file 2: Figure S1. Alignment of the protein sequences of *KCNJ12* in different species. **Figure S2.** Boxplot of reliability of the nine body traits. **Figure S3.** Expression levels of KCNJ12 in 45 tissues. **Figure S4.** The effect of the CNV100’s copy number on the gene expression of *KCNJ12* in muscle tissues. (PDF 678 kb)
