# Supplementary material for: Genome-wide CNV analysis reveals variants associated with growth traits in Bos indicus
Source: BMC Genomics. 2016 Jun 1;17:419. doi: 10.1186/s12864-016-2461-4 (PMC4888316; doi:10.1186/s12864-016-2461-4)
Supplement: Additional file 2: Figure S1. — Alignment of the protein sequences of KCNJ12 in different species. Figure S2. Boxplot of reliability of the nine body traits. Figure S3. Expression levels of KCNJ12 in 45 tissues. Figure S4. The effect of the CNV100’s copy number on the gene expression of KCNJ12 in muscle tissues. (PDF 678 kb) [file 12864_2016_2461_MOESM2_ESM.pdf]

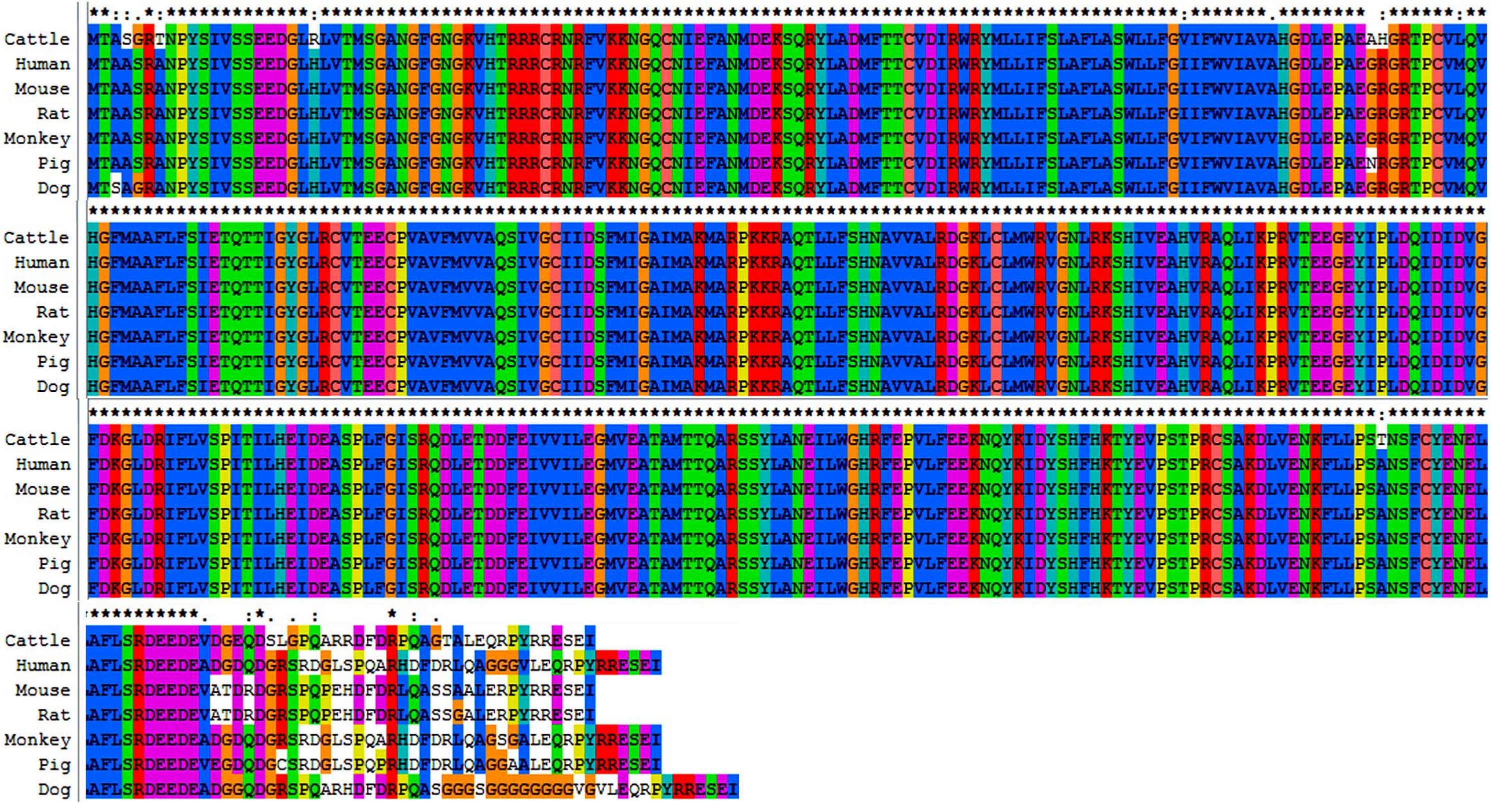
Cattle
Human
Mouse
Rat
Monkey
Pig
Dog

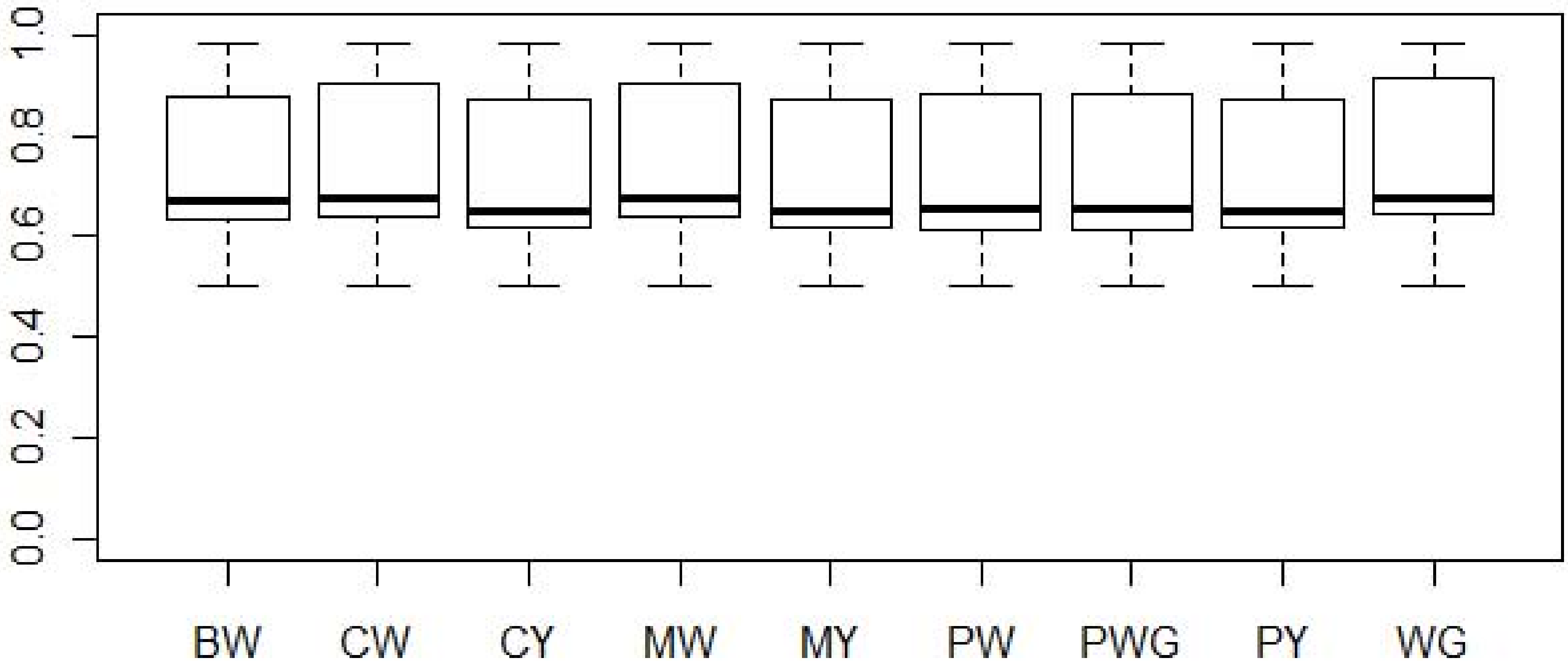
0.0
0.2
0.4
0.6
0.8
1.0
BW
CW
CY
MW
MY
PW
PWG
PY
WG

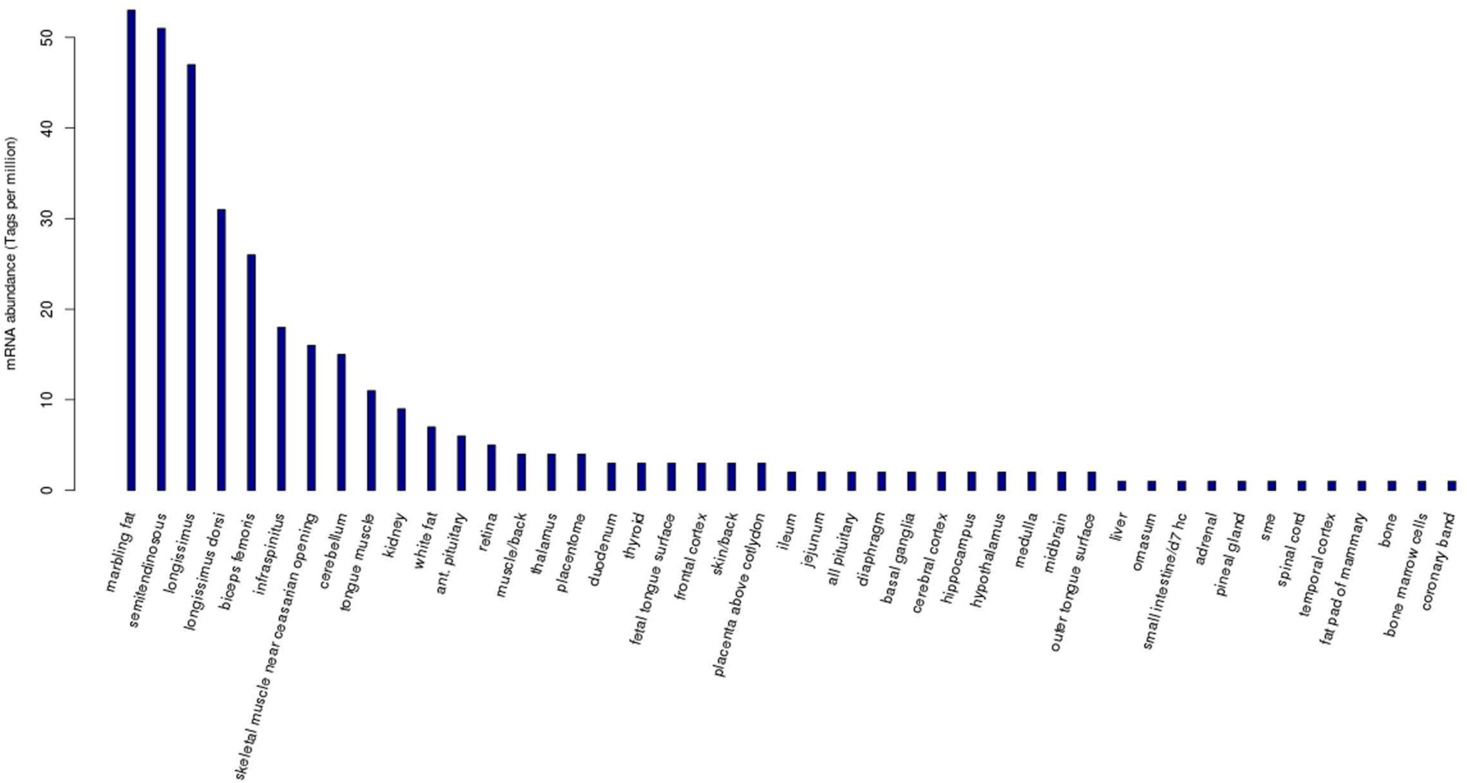

mRNA abundance (Tags per million)
0
10
20
30
40
50
marbling fat
semitendinosous
longissimus
longissimus dorsi
biceps femoris
infraspinitus
skeletal muscle near ceasarian opening
cerebellum
tongue muscle
kidney
white fat
ant. pituitary
retina
muscle/back
thalamus
placentome
duodenum
thyroid
fetal tongue surface
frontal cortex
skin/back
placenta above cotlydon
ileum
jejunum
all pituitary
diaphragm
basal ganglia
cerebral cortex
hippocampus
hypothalamus
medulla
midbrain
outer tongue surface
liver
omasum
small intestine/d7 hc
adrenal
pineal gland
sme
spinal cord
temporal cortex
fat pad of mammary
bone
bone marrow cells
coronary band

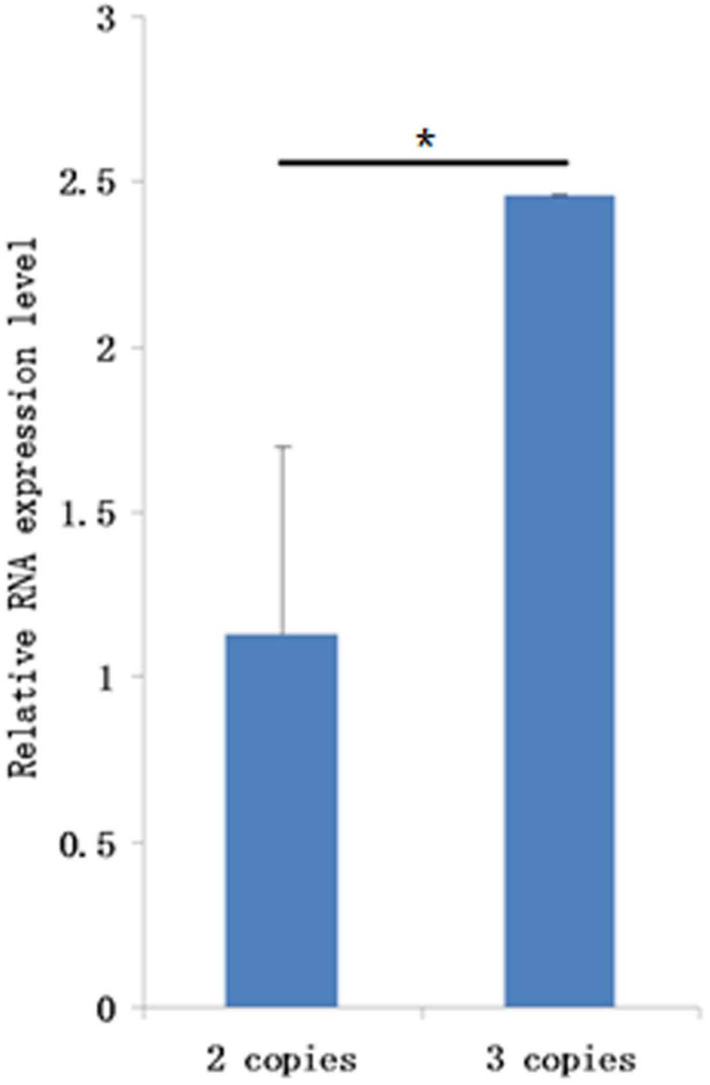

Relative RNA expression level
3
2.5
2
1.5
1
0.5
0
*
2 copies
3 copies
